# Phytochemical Constituent of Devil Weed (*Chromolaena odorata*), Concurrent with Its Antioxidant, α-Glucosidase Inhibitory, and Antibacterial Activity

**DOI:** 10.3390/molecules30214314

**Published:** 2025-11-06

**Authors:** Anastasia Wheni Indrianingsih, Muhammad F. F. Ahla, Anjar Windarsih, Suratno, Tri Wiyono, Eka Noviana, Nurrulhidayah Ahmad Fadzhillah, Ririn Nur Alfiani

**Affiliations:** 1 Research Center for Food Technology and Processing, National Research and Innovation Agency (BRIN), Yogyakarta 55861, Indonesia; anjarwindarsih2@gmail.com (A.W.); nanosan80@gmail.com (S.); triwiyono.mm@gmail.com (T.W.); ririnnalfiani@gmail.com (R.N.A.); 2 Department of Chemistry, Universitas Negeri Malang, Malang 65145, Indonesia; ahlafaiq@gmail.com; 3 Department of Pharmaceutical Chemistry, Faculty of Pharmacy, Universitas Gadjah Mada, Yogyakarta 55281, Indonesia; eka.noviana@ugm.ac.id; 4 International Institute for Halal Research and Training (INHART), International Islamic University Malaysia (IIUM), Kuala Lumpur 50728, Malaysia; nurrulhidayah@iium.edu.my

**Keywords:** *Chromolaena odorata*, α-glucosidase inhibitory, antibacterial, antioxidant, LC-HRMS

## Abstract

This study aimed to investigate the phytochemical constituents of *C. odorata* leaves and stems and to evaluate their antioxidant, total phenol, α-glucosidase, and antibacterial activities. Furthermore, liquid chromatography-high-resolution mass spectrometry (LC–HRMS)-based metabolite profiling combined with principal component analysis (PCA) was applied to correlate metabolite composition with functional activities, providing comprehensive insights into the metabolomic diversity and bioactive differentiation between plant parts. The plant materials were extracted using 70% and 100% ethanol for 24 h. The leaf extract of ethanol 70% (EtOH 70) exhibited the highest antioxidant activity (IC_50_ of 223.33 ± 9.20 µg/mL) and total phenolic content (113.15 mg GAE/g), while the stem EtOH 70% extract showed the strongest antidiabetic activity through α-glucosidase inhibitory activity (78.57%). Although appearing less potent, all extracts showed dose-dependent inhibitory activity, such as *Staphylococcus aureus* (highest value at 9.31 mm), *Escherichia coli* (highest value at 9.92 mm), and *Salmonella typhimurium* (highest value at 9.00 mm). Comparing the plant parts, leaf extracts generally showed more potent activity than stem extracts, particularly evident against *E. coli* (e.g., Leaf EtOH 70% at 5 mg/mL: 9.92 mm vs. Stem EtOH 70%: 7.97 mm). LC-HRMS analysis revealed the presence of phenolics, flavonoids, amino acids, organic acids, and alkaloids. Furthermore, the result indicates that *C. odorata* is a rich source of bioactive compounds with significant antioxidant, α-glucosidase inhibitory, and antibacterial potency. The findings advance existing knowledge beyond earlier phytochemical or single-activity studies, offering a more holistic understanding of *C. odorata*’s therapeutic potential and its relevance for natural product development.

## 1. Introduction

*Chromolaena odorata*, commonly known as devil weed, is a highly invasive plant that poses major ecological and agricultural challenges in tropical regions. Its rapid spread suppresses native flora, alters soil microbial communities, and reduces crop productivity through strong allelopathic effects that inhibit the germination and growth of tropical crops such as mungbean, chilli, and common beans [1,2]. The invasion of *C. odorata* disrupts ecosystem balance by altering soil nutrient dynamics and biodiversity, posing difficulties for sustainable land management [3,4]. However, its biomass has been used for soil fertility restoration, suggesting that appropriate management strategies could balance its ecological threats with potential agronomic benefits [5].

Despite its invasive nature, *C. odorata* is extensively utilized in traditional medicine systems such as Ayurveda, Siddha, and Unani for treating various ailments, including wounds, fever, malaria, and stomach disorders [4,6,7,8]. The leaves of *C. odorata* are commonly crushed to extract juice, which is applied to skin wounds for its haemostatic and anti-inflammatory properties [7,9]. Its rich phytochemical composition, including flavonoids, terpenoids, and phenolic compounds, accounts for its reported antimicrobial, anti-inflammatory, and antioxidant activities [8,10]. In several regions of West Africa, the plant is also valued for improving soil fertility and as a fallow shrub in slash-and-burn agriculture [4,6].

While antioxidant and antidiabetic activities of *C. odorata* have been previously reported, most studies have focused solely on the leaves [11,12,13], often with limited metabolite characterization. There remains a lack of comprehensive comparative analyses of different plant parts and their bioactivities. Therefore, this study aims to evaluate the phytochemical constituents and compare the antioxidant, antidiabetic, and antibacterial properties of the stem and leaf extracts of *C. odorata*. By integrating LC-HRMS-based global metabolite profiling and PCA, this work provides novel insights into the metabolomic diversity and bioactive differentiation between plant parts. The findings advance existing knowledge beyond earlier phytochemical or single-activity studies, offering a more holistic understanding of *C. odorata*’s therapeutic potential and its relevance for natural product development.

## 2. Results and Discussion

### 2.1. Yield of Extraction

Several factors, such as environmental conditions, harvest timing, and biomass composition, influence the yield of extraction from biomass. Biomass yield is significantly affected by precipitation, particularly during the growing season. For instance, May precipitation was found to increase biomass yield, while rainfall in other months had no effect [14]. The timing of biomass harvest plays a crucial role in yield. Harvesting in late summer to early fall or late spring is recommended to maximize yield and minimize moisture content, while winter harvests tend to yield the lowest biomass [15]. The type of biomass, such as switchgrass or native polyculture, and the presence of specific plant species (e.g., forbs) can influence yield. For example, forb cover was a better predictor of biomass yield than warm-season grass in some locations [14]. The presence of extractives in biomass affects its fuel properties. Extractive-free samples showed a significant decrease in ash content, fixed carbon content, and calorific value, indicating that unextracted biomass has better fuel properties [16].

In this study (Table 1), the extraction of *C. odorata* leaves using 70% ethanol had the highest yield of 15.83%, followed by the extraction of *C. odorata* stems using 70% ethanol at 13.53%. The greater yield observed with 70% ethanol compared to 100% ethanol can be attributed to several factors, such as azeotropic behavior and solvent properties. The unique interaction between ethanol and water at specific concentrations can enhance process efficiency [17]. The presence of water in ethanol can alter the solvent properties, such as polarity and viscosity, which can affect the solubility and reactivity of substances [18]. This suggests that a mixture of ethanol and water (70% ethanol) might have more favorable solvent properties compared to pure ethanol. Furthermore, water can improve the solubility of substrates and intermediates, facilitating better conversion rates [19].

### 2.2. Antioxidant Activity

The DPPH (2,2-diphenyl-1-picrylhydrazyl) assay is a widely used method to evaluate the antioxidant activity of various compounds and extracts. This method is based on the reduction in the DPPH radical, which changes color from violet to yellow as an antioxidant neutralizes it [20]. The assay helps in understanding the relationship between the chemical structure of compounds and their antioxidant activity. The DPPH assay is straightforward and does not require complex instrumentation. It can be adapted for various types of samples, including plant extracts, food products, and synthetic compounds [21]. The assay may not distinguish between different antioxidant mechanisms, such as electron transfer and hydrogen atom transfer [22].

Table 2 shows the antioxidant activity of *C. odorata* leaf and stem extracts using the DPPH test. The results obtained showed that the 70% EtOH leaf extract had the highest antioxidant activity, with an IC_50_ value of 223.33 µg/mL, followed by the 100% EtOH leaf extract at 458.99 µg/mL. Meanwhile, *C. odorata* stem extract had lower antioxidant activity than the leaf extract because its IC_50_ value was greater than 1500 µg/mL. In addition, ascorbic acid as positive control had IC_50_ value of 14.31 µg/mL. Several studies have shown that the IC_50_ value of antioxidants using the DPPH method of several extracts has very good antioxidant activity, such as the chloroform extract of *C. odorata* leaves was found to be 310 µg/mL [12], the methanol root extract was reported as 191.68 µg/mL [23], while the n-butanol fraction of *C. odorata* leaves had an IC_50_ value of 33.54 µg/mL [24]. Differences in IC_50_ values from the literature are likely to be due to differences in solvents and extraction methods used [25]. Studies also show that plant origin and harvest conditions can also influence the results of biological activity tests on a plant [26]. While specific data on the DPPH activity of *C. odorata* stem extracts is limited, studies on similar plant parts suggest that the stems also possess significant antioxidant properties due to the presence of bioactive compounds such as phenolics and flavonoids [27]. The high antioxidant activity of *C. odorata* extracts is attributed to their rich phenolic and flavonoid content. These compounds are known for their ability to donate hydrogen atoms or electrons, neutralizing free radicals and preventing oxidative stress [28]. The presence of specific flavonoids such as odoratenin, isosakuranetin, and subscandenin in the methanol extract of *C. odorata* leaves further enhances its antioxidant potential [29].

The beta-carotene bleaching assay is a widely used method for evaluating the antioxidant activity of various substances. This assay measures the ability of antioxidants to prevent the oxidative degradation of beta-carotene in the presence of peroxyl radicals [30]. The assay involves spectrophotometric measurement of beta-carotene concentration changes in a beta-carotene/peroxyl radical system, both with and without the antioxidant. The assay applies to natural extracts, food samples, and commercial antioxidants, providing robust criteria for comparing antioxidant and prooxidant activities [31].

**Figure 1:**
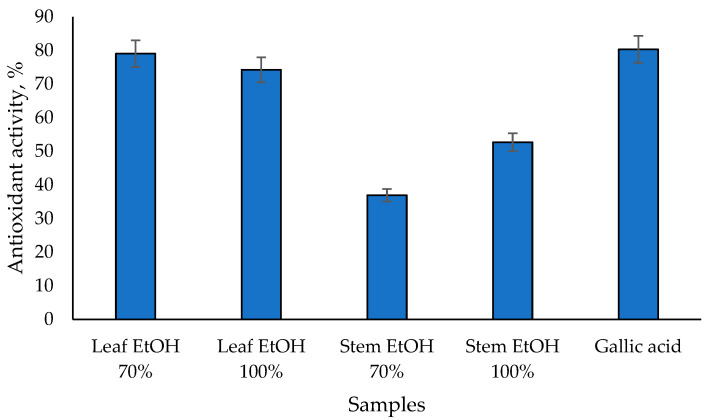
Antioxidant activity of *C. odorata* extracts assessed via *β*-carotene bleaching assay at 1000 µg/mL.

The results of the antioxidant activity test of *C. odorata* leaf and stem extracts using the beta carotene bleaching assay are shown in Figure 1. The 70% EtOH leaf extract has the highest antioxidant activity of 79.05%, followed by the 100% EtOH leaf extract at 74.26%. 100% EtOH and 70% EtOH stem extracts have lower antioxidant activities of 52.72% and 36.96%, respectively. On the other hand, gallic acid as a positive control has the highest value of 80.33%. These results are linear with the antioxidant activity test using the DPPH assay, in which *C. odorata* leaf extract has a higher antioxidant activity than its stem extract. A previous study showed that the ethanol extract of *C. odorata* leaves demonstrated potent antioxidant activity using the beta-carotene bleaching assay, with an IC_50_ value of ≤50 µg/mL [32]. Meanwhile, the ethanol extract of *C. odorata* stems showed vigorous antioxidant activity with an IC_50_ value ranging from 50 to 100 µg/mL [32]. The antioxidant activity observed in the beta-carotene bleaching assay is likely due to the high content of phenolics and flavonoids in the extracts. The total phenolic content and total flavonoid content of *C. odorata* extracts varied significantly, with the leaf extracts showing higher values compared to the stem extracts [32].

Bioactive compounds such as rutin and coumarin are also observed from the LC-HRMS analysis of *C. odorata* (Table 3). Rutin has been shown to have potent free radical scavenging effects in multiple in vitro assays, including DPPH free radical scavenging, lipid peroxidation, and reducing power assays [33]; meanwhile, coumarins exhibit antioxidant activity primarily through free radical scavenging. Both compounds contribute to various health benefits through their antioxidant mechanisms, making them valuable in medicinal and therapeutic applications.

### 2.3. α-Glucosidase Inhibitory Activity

**Figure 2:**
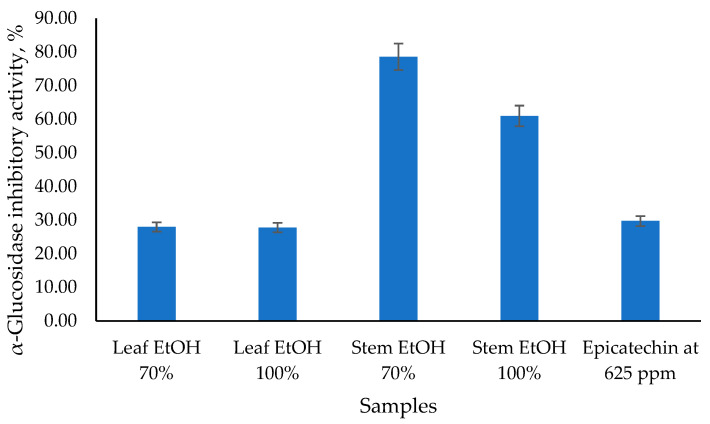
The α-glucosidase inhibitory activity of *C. odorata* leaf and stem using the α-glucosidase inhibitory assay at 1000 µg/mL.

The antidiabetic potential of *C. odorata* has been explored through its α-glucosidase inhibitory activity, as presented in Figure 2, which is a key mechanism in managing diabetes by slowing down carbohydrate digestion and glucose absorption. The results obtained showed that the 70% EtOH stem extract had the highest α-glucosidase inhibitory activity value of 78.57%, followed by the 100% EtOH stem extract at 60.98%. Meanwhile, 70% EtOH and 100% EtOH leaf extracts had similar α-glucosidase inhibitory activity values of 27.97% and 27.77%, respectively. Epicatechin (EC), as a standard, has α-glucosidase inhibitory activity values of 29.74%. Epicatechin has been identified as an α-glucosidase inhibitor, which can help manage postprandial hyperglycemia by reducing the breakdown of carbohydrates into glucose. EC acts as a mixed-type inhibitor, meaning it can bind to both the enzyme and the enzyme-substrate complex, altering the enzyme’s activity [34]. This type of inhibition is beneficial as it can provide a more comprehensive reduction in enzyme activity. Epicatechin (EC), as a standard, has α-glucosidase inhibitory activity values of 29.74%.

Previous study showed that the methanolic leaf extract of *C. odorata* contains several flavonoids and phenolic acids. Among these, isosakuranetin exhibited significant α-glucosidase inhibitory activity with an IC_50_ of 55.99 ± 1.25 μg/mL. Other flavonoids such as 4-O-methylsakuranetin, odoratin, ombuin, and rhamnetin showed weaker inhibitory activities [35]. In diabetic-induced rats, the ethanolic extract of *C. odorata* leaves significantly reduced blood glucose levels and increased insulin levels. This suggests that the extract not only inhibits α-glucosidase but also promotes beta-cell regeneration in the pancreas [36].

Additionally, the extract upregulated the expression of genes involved in glucose metabolism and oxidative stress response, indicating a multifaceted approach to managing diabetes [13]. Another study showed that the ethyl acetate fraction of the flower extract demonstrated potent α-glucosidase inhibitory activity with an IC_50_ of 53.87 ± 0.42 μg/mL, which is comparable to the activity observed in leaf extracts [37]. The ethanolic extract of *C. odorata* leaves was found to upregulate the expression of genes involved in glucose metabolism (Glut2 and glucokinase) and oxidative stress response (Nrf2), while downregulating Keap1, which is a negative regulator of Nrf2. This indicates a multifaceted approach to managing diabetes, including enhancing glucose uptake and reducing oxidative stress [13].

From the LC-HRMS analysis, leaf and steam extract of *C. odorata* had rutin and coumarin (Table 3). These two compounds may play a role in the antidiabetic activity of *C. odorata*. Rutin helps regulate blood glucose levels by enhancing tissue glucose uptake, reducing carbohydrate absorption in the intestines, preserving islet cell function, and boosting insulin secretion [38]; meanwhile coumarin administration significantly reduces plasma glucose levels and glycosylated hemoglobin (HbA1c) in diabetic rats [39].

### 2.4. Antibacterial Activity

The disc diffusion assay revealed that both leaf and stem extracts of *C. odorata*, prepared using either 70% or 100% ethanol, exhibited measurable inhibitory activity against all three tested bacterial pathogens: *S. aureus*, *E. coli*, and *S. typhimurium* (Table 4). A clear concentration-dependent effect was consistently observed for nearly all active extracts, where the mean zone of inhibition decreased significantly (*p* < 0.05) as the extract concentration was reduced from 5 mg/mL to 1.25 mg/mL. For example, leaf extracts (both solvents) against *S. aureus* showed significant stepwise decreases (e.g., Leaf EtOH 70%: 9.31 mm → 8.05 mm → 7.23 mm). This trend highlights the dose-responsive nature of the antibacterial compounds present in the extracts, indicating that higher concentrations yield a greater antimicrobial effect. These results support previous studies that reported that devil weed has anti-pathogenic bacterial activity [40].

While demonstrating activity, the potency of the *C. odorata* extracts was markedly lower than the positive control antibiotic amoxicillin (10 µg), which consistently produced large inhibition zones averaging around 30 mm. Comparing the plant parts, leaf extracts generally showed more potent activity than stem extracts, particularly evident against *E. coli* (e.g., Leaf EtOH 70% at 5 mg/mL: 9.92 mm vs. Stem EtOH 70%: 7.97 mm). The effect of solvent concentration was variable and bacteria-dependent; 100% EtOH sometimes yielded slightly larger zones (e.g., Leaf against *S. aureus* at 2.5 mg/mL: 8.94 mm vs. 8.05 mm for 70%). In comparison, 70% EtOH was sometimes more effective (e.g., Leaf against *E. coli* at 5 mg/mL: 9.92 mm vs. 9.02 mm for 100%). *S. typhimurium* appeared less susceptible overall, with many extracts showing minimal activity (~6 mm) at lower concentrations.

In line with the results of this study, previous research reported that *C. odorata* leaf extract effectively inhibited all strains of *S. suis* bacteria with an MIC of 3.9–62.5 mg/mL [41]. These results confirm the presence of antibacterial principles in *C. odorata* leaves and stems, validating its potential for further investigation, but highlight that its crude extracts are significantly less potent than conventional antibiotics like amoxicillin against these specific pathogens. Referring to the results of metabolite content evaluation, several compounds have antibacterial activity, including chlorinated-quinoline derivatives. Sambavekar et al. [42] synthesized quinoline derivatives that showed antibacterial activity against *S. aureus* (100 μg/mL) equivalent to the activity of ampicillin (10 μg/mL). In addition, chlorogenic acid also showed potent antibacterial activity. Chlorogenic acid (CGA) induces bacterial apoptosis-like death in *E. coli* by depleting intracellular reactive oxygen species (ROS), leading to membrane depolarization and DNA fragmentation [43]. Quinic acid also showed similar activity (MIC 80–120 μg/mL) [44,45]. In addition, flavonoid compounds (rutin, luteolin, and genistein) have also been reported to possess antibacterial activity with varying effectiveness [46,47]. However, further purification or fractionation of the extract appears to be necessary to obtain sufficient extract for good therapeutic effectiveness.

### 2.5. Total Phenolic Content

The Folin–Ciocalteu (FC) assay is a widely used method for determining the total phenolic content (TPC) in various samples, including plant extracts, foods, and beverages. This method is based on the reduction in the Folin–Ciocalteu reagent by phenolic compounds, resulting in a blue complex that can be measured spectrophotometrically. The FC reagent reacts with phenolic compounds in an alkaline medium to form a blue complex, which is measured at 765 nm [48]. Gallic acid is commonly used as a standard, and results are expressed as gallic acid equivalents (GAE) [49].

**Figure 3:**
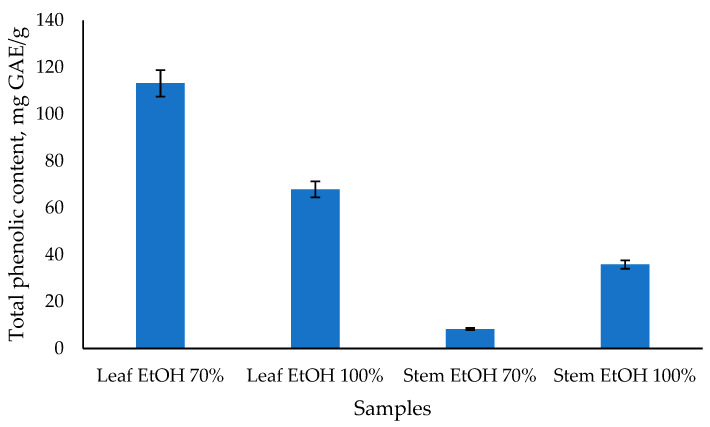
The total phenolic content of *C. odorata* leaf and stem was determined using the Folin-Ciocalteau method.

From Figure 3, it can be seen that the total phenolic content of *C. odorata* leaf extract with 70% EtOH has the highest value, followed by 100% EtOH leaf extract at 113.15 and 67.91 mg GAE/g, respectively. Meanwhile, the stem extract of *C. odorata* has a lower total phenolic content value of 35.85 and 8.33 mg GAE/g, respectively, using 100% EtOH and 70% EtOH solvents. Previous study reported that the highest TPC was found in the aqueous leaf extract of *C. odorata* after 24 h, with a value of 198.02 ± 3.96 mg GAE per gram of extract [50]. Another study reported the TPC of chloroform extract of *C. odorata* leaves to be 242.2 mg/g, which was significantly higher compared to the reference standard gallic acid [12]. The results of the TPC analysis are also linear with those of the antioxidant activity test, indicating that the *C. odorata* leaf extract has a higher value than the stem extract. Several studies found a strong positive correlation between total phenolic content and antioxidant capacity. For instance, one study reported significant correlations using various assays such as ABTS, CUPRAC, FRAP, and DPPH, with correlation coefficients ranging from 0.78 to 0.94 [51]. Another study found a high correlation (R^2^ = 0.814) between total phenolic content and antioxidant capacity measured by DPPH-RSA [52]. Overall, while there is a general trend that higher total phenolic content is associated with greater antioxidant capacity, the strength of this relationship can vary based on the specific plant material, extraction methods, and assays used. This indicates that while phenolics are major contributors to antioxidant activity, other factors and compounds may also play significant roles.

### 2.6. FTIR Spectra

The leaf and stem extracts of *C. odorata* were subjected to FTIR analysis to identify the functional groups of compounds contained in the extracts. FTIR spectroscopy is a powerful analytical technique used to identify functional groups in plant extracts. The method relies on the absorption of infrared radiation by molecular vibrations, which provides a unique spectral fingerprint for different compounds. Moreover, FTIR can help in screening plant extracts for potential medicinal compounds [53].

**Figure 4:**
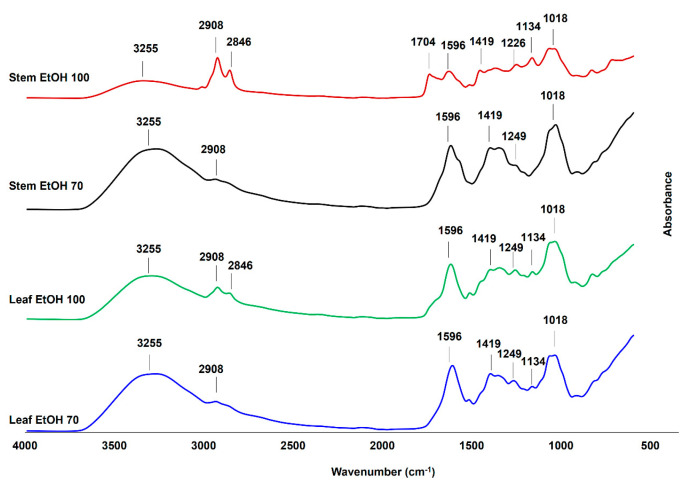
The Fourier transform infrared (FTIR) spectra of leaf and stem extracts of *C. odorata*, extracted using 100% ethanol (EtOH 100) and 70% ethanol (EtOH 70), were measured at a wavenumber range of 4000–600 cm^−1^.

The FTIR spectra of *C. odorata* leaf and stem extracts obtained using 70% and 100% ethanol (Figure 4) revealed the presence of various functional groups, indicating a rich composition of bioactive compounds. Key absorption bands were observed in the region of 4000–600 cm^−1^, corresponding to different molecular vibrations and functional group characteristics. In this study, the identification of FTIR vibration from *C. odorata* stem and leaf extracts is based on previous studies [53,54]. All extracts exhibited a broad peak around 3255 cm^−1^, indicative of O–H stretching vibrations commonly associated with alcohols, phenols, and carboxylic acids. The peaks at 2908 cm^−1^ and 2846 cm^−1^ corresponded to the asymmetric and symmetric vibrations of aliphatic –CH, –CH_2_, and –CH_3_ groups. These bands were more prominent in the EtOH 100% extracts (especially in the stem), implying the presence of lipophilic compounds such as fatty acids and terpenoids, which were better extracted in absolute ethanol [55]. The absorption at 1704 cm^−1^ in the stem EtOH 100% extract suggested the presence of carbonyl groups (C=O), which could be from esters, aldehydes, ketones, or carboxylic acids. This peak was either weak or absent in other extracts, highlighting that 100% ethanol might be more effective in extracting such carbonyl-containing compounds from the stem. The bands at 1596 cm^−1^ and 1419 cm^−1^ were attributed to the vibration of aromatic ring C=C stretching and C–C skeletal vibrations, respectively. These could be characteristic of flavonoids and other aromatic compounds, which were consistently present across all extracts, indicating their abundance in both leaf and stem. The bands at 1134 cm^−1^ and 1018 cm^−1^, present in all extracts, were attributed to the C–O stretching vibration of polysaccharides or secondary alcohols. In addition, the bending vibration of aliphatic –CH, –CH_2,_ and –CH_3_ was observed at around 1226 cm^−1^ and 1249 cm^−1^. Therefore, the FTIR analysis confirmed the presence of various functional groups in *C. odorata* extracts, including hydroxyl, carbonyl, aliphatic, and aromatic compounds that might be from phenolic compounds, organic acids, amino acids and flavonoid compounds. These results were consistent with the result of LC-HRMS metabolites analysis presented in Table 3. These findings support the phytochemical complexity of the plant and its potential pharmacological properties.

### 2.7. LC-HRMS and PCA

Table 3 summarizes major phytochemical constituents in *C. odorata* leaf and stem extracts. Various compounds, including primary metabolites (amino acids, nucleobases, sugars, etc.), secondary metabolites (phenolics, flavonoids, and terpenoids), and lipid/steroid derivatives, were identified in the extracts. Polar compounds such as amino acids, sugar, and sugar derivatives (e.g., glycosides) were extracted due to the relatively high polarity of extracting solvents (i.e., 70% and 100% ethanol). Rutin, a flavonoid glycoside, was more abundant in the 70% ethanolic lead extract, compared with other extracts. The compound has been reported to directly scavenge reactive oxygen species (ROS), inhibit lipid peroxidation, and boost endogenous antioxidants in cells and animal models [56,57]. Other detected flavonoid glycosides include 5,7-dihydroxy-2-(4-hydroxyphenyl)-6,8-bis[3,4,5-trihydroxy-6-(hydroxymethyl)tetrahydro-2H-pyran-2-yl]-4H-chromen-4-one and 5,7-dihydroxy-2-(4-hydroxyphenyl)-4-oxo-4H-chromen-3-yl 6-O-(6-deoxyhexopyranosyl)hexopyranoside.

Phenolic compounds, including flavonoids and coumarins, were also more abundant in the 70% ethanolic leaf extract. This finding is in agreement with results from TPC measurement, in which the leaf extract had the highest phenolic content (113 mg GAE/g extract). Chlorogenic acid and its isomer, neochlorogenic acid, are some antioxidant polyphenols detected in the extract, which are also often found in coffee and tea. Flavonoids detected in the *C. odorata* leaf and stem extracts include rutin, luteolin, isorhamnetin, genistein, hispidulin, padmatin, glycitein, and 5,7-dihydroxy-2-(3-hydroxy-4-methoxyphenyl)-3,6-dimethoxy-4H-chromen-4-one. Many of these compounds have been reported to exhibit antioxidant and anti-inflammatory activity in vitro and in vivo [58,59,60,61]. Coumarin and its derivative, scrophulein, also exhibit several biological activities, including antioxidant, antimicrobial, anti-inflammatory, neuroprotective, antidiabetic, anticonvulsant, and antiproliferative properties [62]. The relatively high abundance of these phenolic compounds likely contributes to the DPPH radical scavenging activity of the 70% ethanolic leaf extract.

In addition to being excellent antioxidants, several phenolic compounds possess moderate to potent antimicrobial activities and can act synergistically with synthetic antibiotics or natural compounds to fight against microbes. Luteolin has been reported to inhibit several methicillin-resistant *S. aureus* (MRSA) isolates with an MIC of 62.5 µg/mL and fluoroquinolone-resistant *Enterococcus* with an MIC of 16 µg/mL [63,64]. A combination of luteolin with ampicillin, oxacillin, and gentamicin can reduce bacterial counts below the detectable limit after 24 h, when tested against MRSA [63]. Other phenolics, including rutin, isorhamnetin, genistein, and hispidulin, have also demonstrated moderate antibacterial activity (MIC > 100 µg/mL) and are more active against Gram-positive bacteria, such as *S. aureus*, MRSA, and *Streptococcus* [65,66,67,68]. The presence of these phenolic compounds in *C. odorata* extracts might explain the more potent antibacterial activity against *S. aureus*, as discussed in the previous section.

Several phytochemical constituents of *C odorata* leaf and stem extracts have previously shown antidiabetic activity. For example, trehalose has been studied in both animals and humans for the prevention and treatment of diabetes [69]. Daily intake of trehalose helped lower postprandial blood glucose levels, potentially due to improved insulin sensitivity and glucose tolerance. Clinical trials have shown that chlorogenic acid can reduce fasting blood glucose levels in individuals with impaired glucose tolerance [70]. Several studies using rodent models reported antidiabetic activity of rutin, luteolin, L-isoleucine, isorhamnetin, genistein, nootkatone, and coumarin derivatives. Rutin lowered glucose levels via multiple potential mechanisms, including the inhibition of carbohydrate-digesting enzymes and modulation of gut microbiota [71]. Similarly, luteolin also inhibits the enzymes (α-glucosidase and DPP-4) and enhances insulin secretion by protecting the pancreatic cells [72]. Improved insulin sensitivity and pancreatic β-cell survival were also found in studies using isorhamnetin, genistein, hispidulin, and nootkatone [73,74,75]. In contrast, L-isoleucine’s antidiabetic effect is independent of insulin. It decreases blood glucose by increasing glucose uptake by skeletal muscle [76].

*C. odorata* leaf and stem ethanolic extracts contain various phytochemicals that exert biological activities such as antioxidant, anti-inflammatory, antidiabetic, and antimicrobial. The abundance of the compounds was affected by the part of the plant and the composition of the extracting solvents (i.e., polar compounds and phenolics mainly were extracted from leaves using 70% ethanol). Understanding the relationship between extraction conditions, phytochemical constituents, and their biological activity can provide directions for phytochemical-targeted extraction for various medicinal applications.

**Figure 5:**
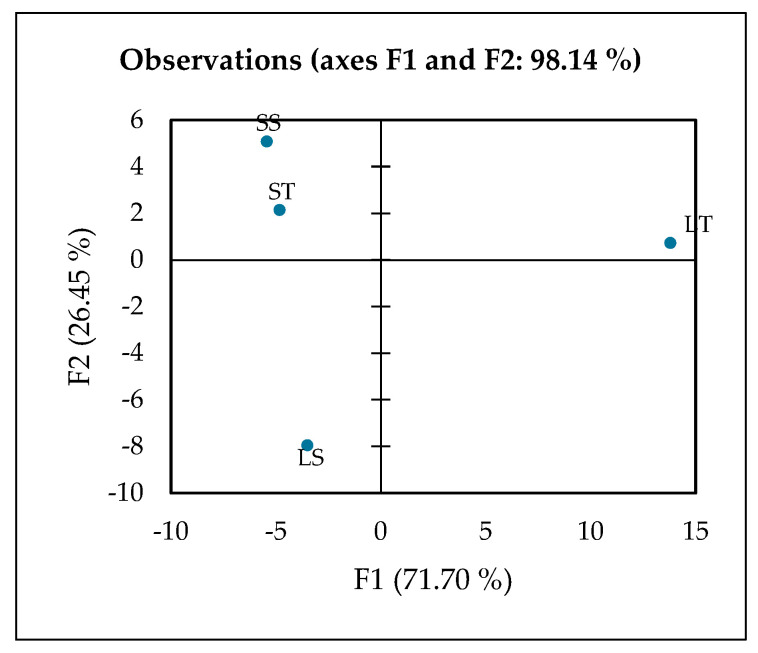
PCA plot observed by XLSTAT analysis, LT (Leaf 70% ethanol extract), LS (Leaf 100% ethanol extract), ST (Stem 70% ethanol extract), and SS (Stem 100% ethanol extract).

The contribution of extraction using 70% or 100% ethanol in leaf or stem samples was further evaluated using XLSTAT. XLSTAT is a practical tool for performing PCA, offering a user-friendly interface and comprehensive analysis capabilities. The first two principal components (F1 and F2) accounted for a cumulative variance of 98.14%, indicating that the two-dimensional PCA plot provides an almost complete representation of the overall variability within the dataset. The PCA revealed that F1 (71.70%) serves as the primary axis of separation, explaining the majority of the total variance and therefore representing the most important source of metabolic differences among the extracts (Figure 5). Along this axis, the leaf 70% ethanol extract (LT) is clearly distinguished from all other groups, occupying a far-positive position on the F1 score plot. This distinct clustering suggests that LT possesses a unique metabolic profile, heavily influenced by the solvent polarity and the tissue type, resulting in a selective enrichment of phenolic metabolites.

The variables driving F1 are dominated by phenolics and flavonoids, including hesperetin, gallic acid, luteolin, rutin, salicylic acid, and phenylglyoxylic acid. These compounds are well-known secondary metabolites in plants, acting as defense molecules and providing strong antioxidant capacity [77]. For instance, gallic acid and rutin are potent radical scavengers that contribute to oxidative stress protection [78,79], while luteolin and hesperetin possess anti-inflammatory and vasoprotective properties [80,81]. Salicylic acid, beyond its role as a plant signaling molecule, is the natural precursor to aspirin, linking LT to potential anti-inflammatory benefits [82]. The simultaneous presence of multiple bioactive phenolics highlights the chemical richness of LT and suggests a synergistic contribution to its biological potency [83].

The metabolic interpretation of this pattern indicates that LT is the most phenolic-rich and functionally potent extract among the tested samples. The strong positive loadings of these phenolic and flavonoid compounds on F1 confirm that phenolic composition is the dominant factor separating LT from the stem extracts and from the 100% ethanol leaf extract (LS). This observation also demonstrates that 70% ethanol is an effective solvent system for recovering phenolic compounds from leaves, likely due to the balance between water and ethanol that maximizes the extraction of mid-polarity phytochemicals [84,85]. Consequently, LT not only serves as a chemically distinct sample but also as a promising candidate for functional food or nutraceutical applications aimed at delivering antioxidant and anti-inflammatory benefits [86,87].

F2 (26.45%) represents the secondary axis of separation, which distinguishes the leaf 100% ethanol extract (LS) from the stem-derived extracts (ST and SS). LS is strongly correlated with compounds such as adenine, guanine, L-isoleucine, L-phenylalanine, α,α-trehalose, N-acetylvaline, and amines, reflecting enrichment in primary metabolites including amino acids, nucleotides, and sugars. These molecules play crucial roles in plant energy metabolism, protein biosynthesis, and stress response [88,89]. The pattern suggests that absolute ethanol extraction favors the extraction of lower-polarity or nitrogen-containing compounds rather than polyphenols, consistent with reports that solvent polarity critically influences metabolite recovery [84,85]. Thus, although LS is less enriched in phenolics compared to LT, it provides a distinct biochemical profile centered on primary metabolism, making it metabolically complementary to phenolic-rich extracts. The positioning of LS along F2 underscores the importance of solvent strength in shaping extract composition.

Stem 70% ethanol extract (ST) is located closer to the central area of the PCA plot with a slight contribution from F3, indicating more subtle metabolic differentiation. Compounds most associated with ST include sphinganine, N,N-diethyldodecanamide, and N-benzylformamide, which are primarily lipid derivatives and amides. These metabolites are characteristic of membrane structure, signaling lipids, and nitrogen metabolism, reflecting tissue-specific biochemistry of stems compared to leaves [90,91]. The ST profile, therefore, emphasizes the structural and metabolic functions of stem tissue, which typically contains more lipids and amine derivatives than leaves, further highlighting tissue-driven metabolic variation.

Stem 100% ethanol extract (SS) occupies an intermediate position between LT and LS on the PCA score plot, suggesting a mixed metabolic profile. It shares some F1-driven phenolics with LT and exhibits specific F2-associated amino acids and sugars similar to those of LS. This intermediate positioning implies that SS contains both polyphenols and primary metabolites, reflecting a balanced extraction outcome rather than a specialized profile. Such hybrid compositions have been observed in metabolomics studies when solvents extract a broad polarity range of compounds [92]. Functionally, SS may represent a bridge extract, capture characteristics of both leaf and stem tissues, and demonstrate how solvent choice and plant part interact to determine final metabolite composition.

The 2D PCA effectively summarizes the main patterns and relationships in the data, allowing clear visualization of sample clustering and differentiation based on their underlying metabolic profiles. The distinct clustering observed in the PCA score plot indicates a strong influence of both tissue type and solvent polarity on metabolite extraction. The 70% ethanol leaf extract (LT) was clearly separated from the aqueous and stem extracts along the first principal component, suggesting a selective enrichment of ethanol-soluble metabolites such as phenolics, flavonoids, and other semi-polar secondary compounds. In contrast, the aqueous leaf (LS) and stem (ST and SS) extracts clustered together, reflecting a predominance of hydrophilic primary metabolites like sugars, amino acids, and organic acids. This separation highlights preferential solvent–metabolite interactions, where the intermediate polarity of ethanol facilitates broader metabolite recovery compared to water alone. Biologically, this distinction implies that the ethanol extract may possess enhanced bioactive potential, supporting its relevance for functional food, nutraceutical, or antioxidant applications.

## 3. Materials and Methods

### 3.1. Materials and Chemicals

*C. odorata* leaves and stems were taken from the Gunungkidul area, Yogyakarta Province, Indonesia, in April 2021. The voucher specimen was identified and deposited in the Research Center for Food Technology and Processing (PRTPP BRIN), Yogyakarta, Indonesia. 2,2-Diphenyl-1-picrylhydrazyl (DPPH), alpha-glucosidase enzyme, ascorbic acid, gallic acid, and Folin–Ciocalteau reagent were purchased from Sigma-Aldrich (Tokyo, Japan).

### 3.2. Preparation of C. odorata Extracts

Dried *C. odorata* leaf and stem were macerated using 70% and 100% ethanol (EtOH, 1:10 *w*/*v*)—the extraction process was conducted for 24 h at 27 °C. After filtration and evaporation, the extracts of *C. odorata* leaf and stem were stored at 4 °C until further analysis. The flowchart of experiments was presented in Figure S1.

### 3.3. Antioxidant Assay

#### 3.3.1. DPPH Assay

The DPPH assay was conducted according to a prior study with slight modification [93]. Briefly, the sample was reacted with DPPH (1.01 mM) for 30 min at room temperature in the dark condition. The absorbance of the final solution was measured using an ELISA reader (Thermo Scientific, Waltham, MA, USA) at 517 nm. Ascorbic acid was used as the positive control. The antioxidant assay was conducted in triplicate. The antioxidant activity of *C. odorata* extracts was calculated using the following Equation (1):

(1) $$\mathrm{DPPH}\;\mathrm{scavenging}\;\mathrm{activity}\;(\%)=(\frac{A_{0}-A_{1}}{A_{0}}\;\times \;100)$$

where A_0_ and A_1_ are absorbances of the DPPH solution in the absence and presence of the extract, respectively.

#### 3.3.2. β-Carotene-Bleaching Assay

The antioxidant action of *C. odorata* extracts was evaluated using the β-carotene-linoleate test according to a previous study [94]. Briefly, β-carotene reagent (consisting of 0.2 mg of β-carotene, 1 mL of linoleic acid, 20 mg of Tween 40, and 5 mL of distilled water) was reacted with the extract (1000 µg/mL in methanol). The mixture was incubated at 50 °C for 120 min, and the absorbance was measured at 470 nm using an ELISA reader (Thermo Scientific, Waltham, MA, USA) at 0 and 120 min. Ascorbic acid (1000 µg/mL) was used as the positive control. The antioxidant activity was calculated using the following Equation:

$$Antioxidant\;activity(\%)=100[1-\frac{\left(As0-As120\right)}{\left(Ab0-Ab120\right)}]$$

where As0 and As120 are the absorbance of β-carotene in the presence of extract at 0 min and 120 min, respectively, while Ab0 and Ab120 is the absorbance of β-carotene without the extract at 0 min and 120 min, respectively.

### 3.4. α-Glucosidase Inhibitory Assay

The antidiabetic activity of *C. odorata* extract was performed according to a prior study [93]. Briefly, the samples (20 μL, 1000 ppm, in 20% dimethyl sulfoxide) was reacted with α-glucosidase enzyme (40 μL). After 5 min of incubation at 37 °C, 4-nitrophenyl α-D glucopyranoside (40 μL, 3 mM) in a phosphate buffer solution at pH 7 was added to the solution, and the incubation continued for 20 min at 37 °C. Sodium carbonate (0.2 M) was added to stop the reaction, and the absorbance of the final solution was measured at 405 nm using an ELISA reader (Thermo Scientific, USA).

### 3.5. Total Phenolic Content (TPC) Analysis

The total phenolic content of *C. odorata* extract was evaluated using the Folin–Ciocalteu reagent [94]. Briefly, the samples (10 μL, 1000 ppm) was reacted with Folin–Ciocalteu reagent (50 μL), Na_2_CO_3_ (20%, 150 μL), and water (790 μL). After 2 h of incubation at room temperature, the absorbance of the final solution was measured at 765 nm using an ELISA reader (Thermo Scientific, USA). The analysis was conducted in triplicate.

### 3.6. Antibacterial Study

The ability of *C. odorata* extracts to inhibit or kill the bacteria was tested using the agar disc diffusion method [94]. The extracts were tested against *S. aureus*, *E. coli*, and *S. typhimurium.* Concisely, extract (1000 ppm) was put into a circular disk with a diameter of 6 mm. The disks were placed onto the MHA medium plate containing 10^8^ CFU/mL bacteria (equivalent to a 0.5 Mc Farland standard). A six mm-diameter amoxicillin disc (positive control, 10 µg in a disc) and sterile distilled water (negative control) were also placed onto the medium plate. The plates were incubated for 24 h at 37 °C. The diameter of inhibition zones was then measured and used to assess the bacterial activity of the samples and control. Measurement results were reported as the average of three independent observations.

### 3.7. FTIR Study

All extracts of *C. odorata* were evaluated for their functional group using FTIR analysis according to a previous research [95]. FTIR spectra were recorded at a resolution of 4 cm with 32 scans across the 4000–500 cm^−1^ range.

### 3.8. LC-HRMS Study

The metabolite constituents of *C. odorata* extracts were evaluated using LC-HRMS (Thermo Scientific™ Vanquish™ UHPLC Binary Pump and Orbitrap high-resolution mass spectrometry; Thermo Scientific, Waltham, MA, USA) according to the literature [96]. Analysis was performed using an Accucore Phenyl-hexyl column (100 mm × 2.1 mm × 2.6 μm) (Thermo Scientific, Waltham, MA, USA) maintained at 40 °C. A total of 5 μL of samples was injected and eluted using mobile phase consisted of water (A) and methanol (B) both containing 0.1% formic acid with the flow rate of 0.30 mL/min. A gradient elution technique was used programmed as follows: 0–5 min (5% B), 5–16 min (5% B to 90% B), 16–20 min (90% B), and 20–25 min (90% B to 5% B). The sheath gas flow rate in LC-HRMS instrument was set at 32 arbitrary unit (AU) with auxiliary gas flow rate of 8 AU. The compounds were detected in both positive and negative ionization modes with scan range of 66.7–1000 m/z and resolution of 70,000 for MS1 and 17,500 for MS2. The capillary temperature was set at 320 °C during the analysis.

### 3.9. Statistical Analysis

The biological activity of *C. odorata* extract was statistically examined using one-way analysis of variance (ANOVA) followed by Duncan’s test [93]. PCA was performed utilizing XLSTAT Student 2025.1.3.1431 Software (Lumivero, France) [97].

## 4. Conclusions

This study provides comprehensive evidence that *Chromolaena odorata* possesses a wide range of pharmacologically active compounds contributing to its significant antidiabetic, antioxidant, and antibacterial activities. The 70% ethanol leaf extract demonstrated the most substantial antioxidant potential, correlating with its high phenolic content. In comparison, the 70% ethanol stem extract showed superior α-glucosidase inhibitory activity, indicating promising antidiabetic effects. Although the pathogenic antibacterial activity appears to be less potent, there is still a dose-dependent relationship to the inhibition of *S. aureus*, *E. coli*, and *S. typhimurium* that requires further research or fractionation. The LC-HRMS analysis has facilitated the identification of numerous bioactive compounds, including phenolics, flavonoids, alkaloids, and organic acids, providing a deeper understanding of the chemical profile of *C. odorata*. The findings justify the traditional use of *C. odorata* and indicate it is a promising candidate for developing plant-based treatments to lower blood sugar, particularly for conditions related to oxidative stress.

## Figures and Tables

**Table 1:** The extraction yield of *C. odorata* leaf and stem by the maceration method using ethanol 70% and ethanol 100%.

Sample	Solvent	Yield (%)
Leaf	Ethanol 70%	15.83 ± 1.32 ^d^
Leaf	Ethanol 100%	11.02 ± 1.10 ^b^
Stem	Ethanol 70%	13.53 ± 0.51 ^c^
Stem	Ethanol 100%	10.22 ± 0.54 ^a^

Means followed by different letters in the same column indicate significant differences (*p* < 0.05) according to ANOVA and Duncan’s test.

**Table 2:** Antioxidant activity of *C. odorata* extracts assessed via DPPH assay.

Sample	IC_50_, µg/mL	% Radical Scavenging Activity at 400 µg/mL
Leaf EtOH 70%	223.33 ± 9.20 ^b^	80.36 ± 2.52
Leaf EtOH 100%	458.99 ± 3.51 ^c^	55.28 ± 3.58
Stem EtOH 70%	2706.76 * ± 18.32 ^e^	7.65 ± 0.65
Stem EtOH 100%	1535.86 * ± 13.71 ^d^	13.56 ± 1.12
Ascorbic acid	14.31 ± 0.09 ^a^	

Means followed by different letters in the same column indicate significant differences (*p* < 0.05) according to ANOVA and Duncan’s test. * IC_50_ value was estimated by extrapolation.

**Table 3:** The constituents of *C. odorata* leaf & stem were analyzed using LC-HRMS analysis.

No	Name	Formula	Calc. MW	RT [Min]	Leaf 70% (% Relative)	Leaf 100% (% Relative)	Stem 70% (% Relative)	Stem 100% (% Relative)
1	α,α-Trehalose	C_12_H_22_ O_11_	342.1155	0.824	0.002	0.180	1.929	2.163
2	Adenosine	C_10_H_13_ N_5_ O_4_	267.0956	0.828	0.002	0.068	3.134	5.700
3	D-(-)-Quinic acid	C_7_H_12_ O_6_	192.0627	0.83	0.029	0.041	1.741	0.550
4	3,4,5-trihydroxycyclohex-1-ene-1-carboxylic acid	C_7_H_10_ O_5_	174.0522	0.861	0.004	0.041	0.081	0.100
5	Nicotinic acid	C_6_H_5_ N O_2_	123.0318	1.031	0.001	8.585	0.785	0.629
6	Tyramine	C_8_H_11_ N O	137.0836	1.109	0.001	11.313	0.182	0.100
7	Adenine	C_5_H_5_ N_5_	135.054	1.192	0.0001	1.928	0.502	0.546
8	L-Isoleucine	C_6_H_13_ N O_2_	131.0942	1.216	0.010	0.045	14.071	14.741
9	Guanine	C_5_H_5_ N_5_ O	151.0487	1.261	0.001	2.326	1.697	1.470
10	(2S)-4-Methyl-2-({[(3S,4S,5R)-2,3,4-trihydroxy-5-(hydroxymethyl)tetrahydro-2-furanyl]methyl}amino)pentanoic acid	C_12_H_23_ N O_7_	293.1467	1.329	0.0006	5.307	1.274	1.469
11	4-Hydroxy-6-methyl-2-pyrone	C_6_H_6_ O_3_	126.0312	1.638	0.0002	8.385	35.884	44.544
12	L-Phenylalanine	C_9_H_11_ N O_2_	165.0785	1.961	0.003	0.018	2.675	3.344
13	trans-3-Indoleacrylic acid	C_11_H_9_ N O_2_	187.0625	3.108	0.003	0.015	2.148	4.014
14	Neochlorogenic acid	C_16_H_18_ O_9_	354.0947	3.449	0.001	3.281	0.114	0.068
15	Chlorogenic acid	C_16_H_18_ O_9_	354.0941	5.327	0.014	0.040	1.268	0.792
16	2,3-Dihydro-1-benzofuran-2-carboxylic acid	C_9_H_8_ O_3_	164.0467	5.775	0.001	0.004	0.349	0.330
17	5,7-Dihydroxy-2-(4-hydroxyphenyl)-6,8-bis [3,4,5-trihydroxy-6-(hydroxymethyl)tetrahydro-2H-pyran-2-yl]-4H-chromen-4-one	C_27_H_30_ O_15_	594.1571	7.076	0.003	8.308	0.277	0.099
18	{(1R,2R)-2-[(2Z)-5-(Hexopyranosyloxy)-2-penten-1-yl]-3-oxocyclopentyl}acetic acid	C_18_H_28_ O_9_	388.1728	7.193	0.005	0.034	2.439	2.960
19	(1S,3R,4R,5R)-1,3,4-trihydroxy-5-{[(2E)-3-(4-hydroxy-3-methoxyphenyl)prop-2-enoyl]oxy}cyclohexane-1-carboxylic acid	C_17_H_20_ O_9_	368.1102	7.245	0.001	12.659	0.089	0.063
20	2-(2,4-dihydroxyphenyl)-3,5,7-trihydroxy-4H-chromen-4-one	C_15_H_10_ O_7_	302.0417	7.423	0.0004	5.229	0.030	0.012
21	Rutin	C_27_H_30_ O_16_	610.1519	8.618	0.010	0.037	1.369	0.699
22	Coumarin	C_9_H_6_ O_2_	146.0364	8.862	0.002	0.031	0.648	0.355
23	2,4,6-Trihydroxy-2-(4-hydroxybenzyl)-1-benzofuran-3(2H)-one	C_15_H_12_ O_6_	288.063	8.916	0.011	0.345	0.963	0.981
24	4-(4-benzhydrylpiperidino)-8-chloro-2-(trifluoromethyl)quinoline	C_28_H_24_ Cl F_3_ N_2_	480.1597	9.337	0.001	0.006	0.144	0.163
25	5,7-Dihydroxy-2-(4-hydroxyphenyl)-4-oxo-4H-chromen-3-yl 6-O-(6-deoxyhexopyranosyl)hexopyranoside	C_27_H_30_ O_15_	594.1579	9.487	0.002	9.186	0.351	0.233
26	Padmatin	C_16_H_14_ O_7_	318.0725	10.627	0.023	0.979	1.025	0.897
27	Nootkatone	C_15_H_22_ O	218.1662	10.909	0.002	0.165	1.052	0.940
28	N,N-Dimethyldecylamine N-oxide	C_12_H_27_ N O	201.2084	11.59	0.001	0.108	1.645	1.531
29	Luteolin	C_15_H_10_ O_6_	286.0475	11.649	0.003	0.082	0.029	0.022
30	(2R,3R)-3,5-dihydroxy-2-(4-hydroxyphenyl)-7-methoxy-3,4-dihydro-2H-1-benzopyran-4-one	C_16_H_14_ O_6_	302.0779	11.694	0.045	1.568	2.660	1.195
31	Isorhamnetin	C_16_H_12_ O_7_	316.0575	11.807	0.023	0.537	0.096	0.067
32	Genistein	C_15_H_10_ O_5_	270.0524	11.862	0.001	0.038	0.040	0.014
33	(6,6-Dimethylbicyclo[3.1.1]hept-2-yl)methyl 6-O-[(2R,3R,4R)-3,4-dihydroxy-4-(hydroxymethyl)tetrahydro-2-furanyl]-β-D-glucopyranoside	C_21_H_36_ O_10_	448.2302	11.981	0.0002	1.915	0.768	0.850
34	Hispidulin	C_16_H_12_ O_6_	300.0629	12.028	0.002	0.048	0.066	0.019
35	Scrophulein	C_17_H_14_ O_6_	314.0778	12.905	0.018	0.835	0.902	0.184
36	5,7-dihydroxy-2-(3-hydroxy-4-methoxyphenyl)-3,6-dimethoxy-4H-chromen-4-one	C_18_H_16_ O_8_	360.0835	12.969	0.003	0.108	0.079	0.038
37	N,N-Diethyldodecanamide	C_16_H_33_ N O	255.2553	13.266	0.001	0.062	1.386	1.061
38	Corchorifatty acid F	C_18_H_32_ O_5_	328.2242	13.274	0.001	0.026	0.528	0.189
39	Glycitein	C_16_H_12_ O_5_	284.0675	13.549	0.035	1.643	1.808	0.210
40	19-Norandrostenedione	C_18_H_24_ O_2_	272.1765	14.024	0.005	0.178	0.171	0.023
41	12-oxo Phytodienoic Acid	C_18_H_28_ O_3_	292.2032	15.665	99.710	4.762	5.310	0.257
42	Bis(2-ethylhexyl) amine	C_16_H_35_ N	241.2761	15.716	0.001	1.640	1.424	1.411
43	Octadecanamine	C_18_H_39_ N	269.3075	16.479	0.003	7.457	4.171	3.000
44	Stearamide	C_18_H_37_ N O	283.2866	18.355	0.014	0.437	2.696	1.967

Note: MW = molecular weight; RT = retention time.

**Table 4:** The antibacterial activity of *C. odorata* leaf and stem using the disc diffusion method.

Sample	Bacteria	Inhibition Zone (mm)		
		5 mg/mL	2.5 mg/ml	1.25 mg/mL
Leaf EtOH 70%	*S. aureus*	9.31 ± 0.61 ^c^	8.05 ± 0.22 ^b^	7.23 ± 0.23 ^a^
Leaf EtOH 100%		9.31 ± 0.73 ^c^	8.94 ± 0.71 ^d^	7.41 ± 0.56 ^a^
Stem EtOH 70%		9.25 ± 0.74 ^b^	7.48 ± 0.64 ^a^	7.43 ± 0.55 ^a^
Stem EtOH 100%		8.03 ± 0.25 ^a^	8.19 ± 0.63 ^c^	7.36 ± 0.53 ^a^
Amoxycillin 10 µg		30.25 ± 2.10 ^d^	29.30 ± 1.70 ^e^	30.52 ± 2.40 ^b^
Leaf EtOH 70%	*E. coli*	9.92 ± 0.46 ^b^	8.33 ± 0.53 ^c^	6.02 ± 0.46 ^a^
Leaf EtOH 100%		9.02 ± 0.62 ^b^	8.69 ± 0.67 ^c^	6.87 ± 0.63 ^b^
Stem EtOH 70%		7.97 ± 0.57 ^a^	6.02 ± 0.55 ^a^	6.02 ± 0.58 ^a^
Stem EtOH 100%		7.79 ± 0.75 ^a^	7.57 ± 0.73 ^b^	6.92 ± 0.44 ^b^
Amoxycillin 10 µg		29.92 ± 1.71 ^c^	29.44 ± 1.31 ^d^	29.86 ± 1.16 ^c^
Leaf EtOH 70%	*S. typhimurium*	6.08 ± 0.65 ^a^	6.02 ± 0.56 ^a^	6.01 ± 0.20 ^a^
Leaf EtOH 100%		7.50 ± 0.53 ^b^	7.24 ± 0.52 ^b^	6.00 ± 0.00 ^a^
Stem EtOH 70%		7.31 ± 0.62 ^b^	6.02 ± 0.63 ^a^	6.02 ± 0.01 ^a^
Stem EtOH 100%		8.00 ± 0.57 ^c^	6.15 ± 0.42 ^a^	6.02 ± 0.01 ^a^
Amoxycillin 10 µg		30.17 ± 1.17 ^d^	28.29 ± 1.22 ^c^	28.57 ± 1.21 ^b^

Means followed by different letters in the same column indicate significant differences (*p* < 0.05) according to ANOVA and Duncan’s test.

## Data Availability

Data will be made available upon request.

## Supplementary Materials

The following supporting information can be downloaded at: https://www.mdpi.com/article/10.3390/molecules30214314/s1, Figure S1: Flow chart about the experimental process.
