# Supplementary figures and images for: Phytochemical Constituent of Devil Weed (Chromolaena odorata), Concurrent with Its Antioxidant, α-Glucosidase Inhibitory, and Antibacterial Activity
Source: Molecules. 2025 Nov 6;30(21):4314. doi: 10.3390/molecules30214314 (PMC12608794; doi:10.3390/molecules30214314)

## Supplementary Information

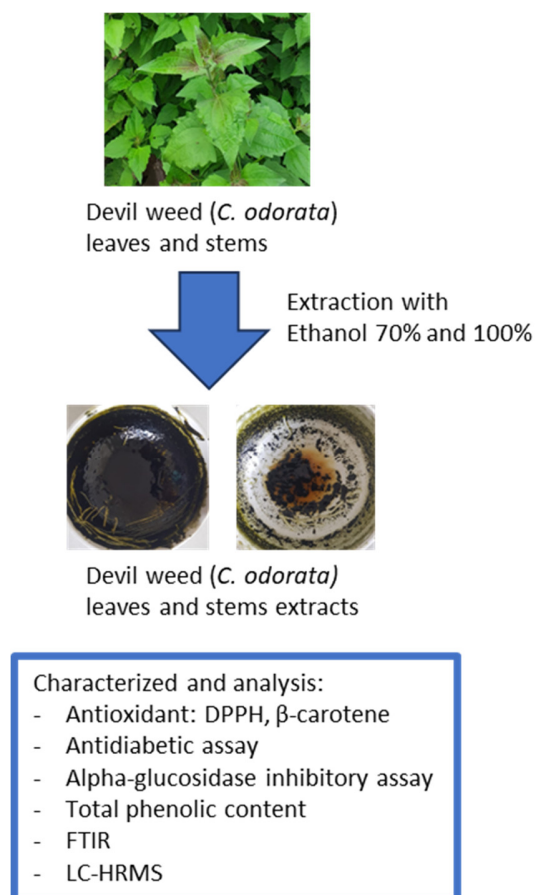

Figure S1. Flow chart about the experimental process

Supplement: Supplementary file 1 [file molecules-30-04314-s001.zip › molecules-3946374-supplementary.pdf]
